# Supplementary material for: Non-invasive human skin transcriptome analysis using mRNA in skin surface lipids
Source: Commun Biol. 2022 Mar 9;5:215. doi: 10.1038/s42003-022-03154-w (PMC8907185; doi:10.1038/s42003-022-03154-w)
Supplement: Supplementary file 2 — Supplementary Information [file 42003_2022_3154_MOESM2_ESM.pdf]

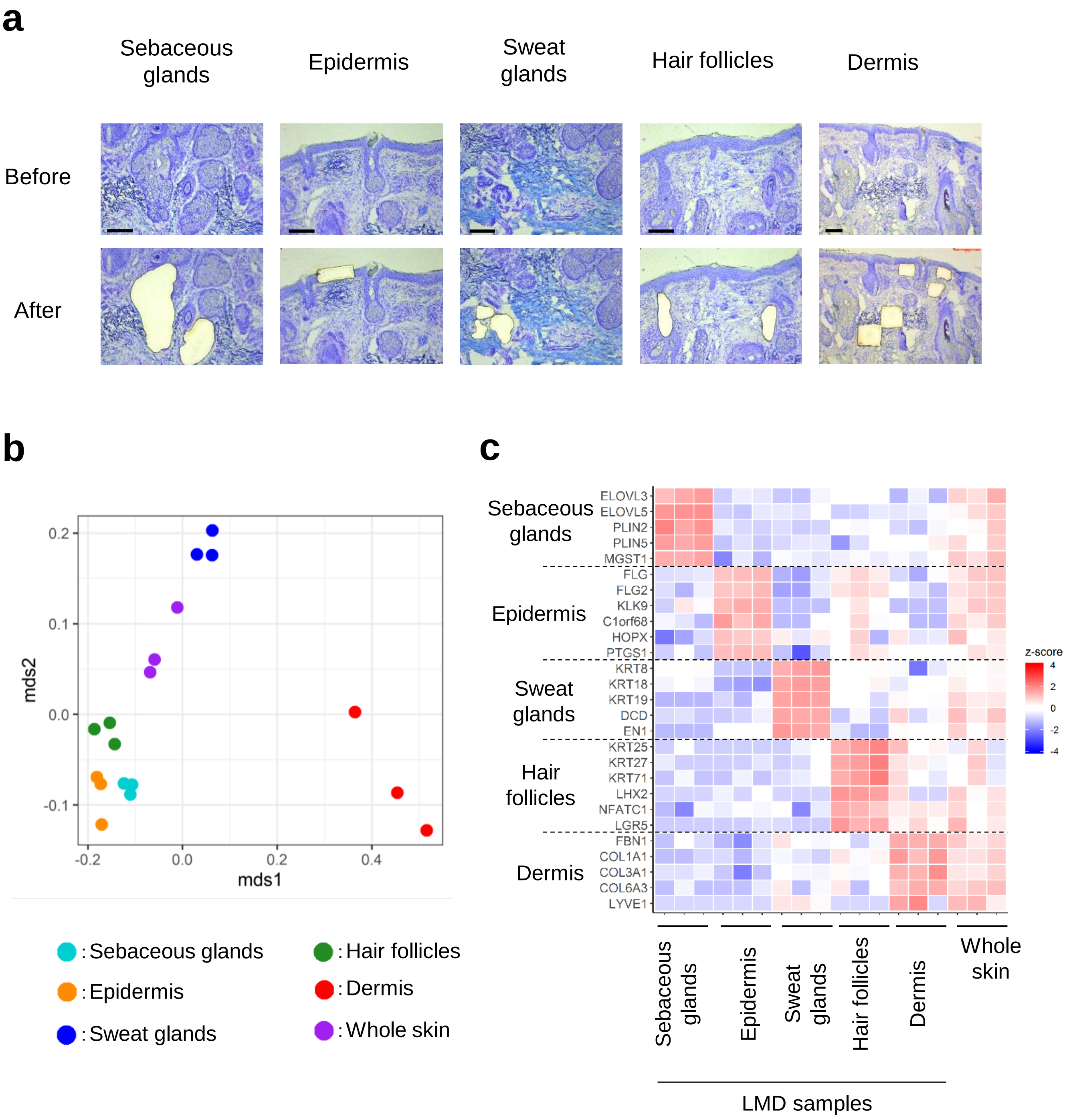

Supplementary Figure 1. Similarity in mRNA expression profiles of SSL-RNA and each skin region.

(a) Tissue images of human sebaceous glands, epidermis, sweat glands, hair follicles, and dermis before and after LMD. Bar: 200 μm. (b) MDS analysis using the expression profile of each region and whole skin, n = 3. (c) mRNA expression profile of LMD samples and whole skin. The heatmap shows z-transformed expression values of marker genes in each LMD sample and whole skin, n = 3. SSL, skin surface lipids; LMD, laser microdissection

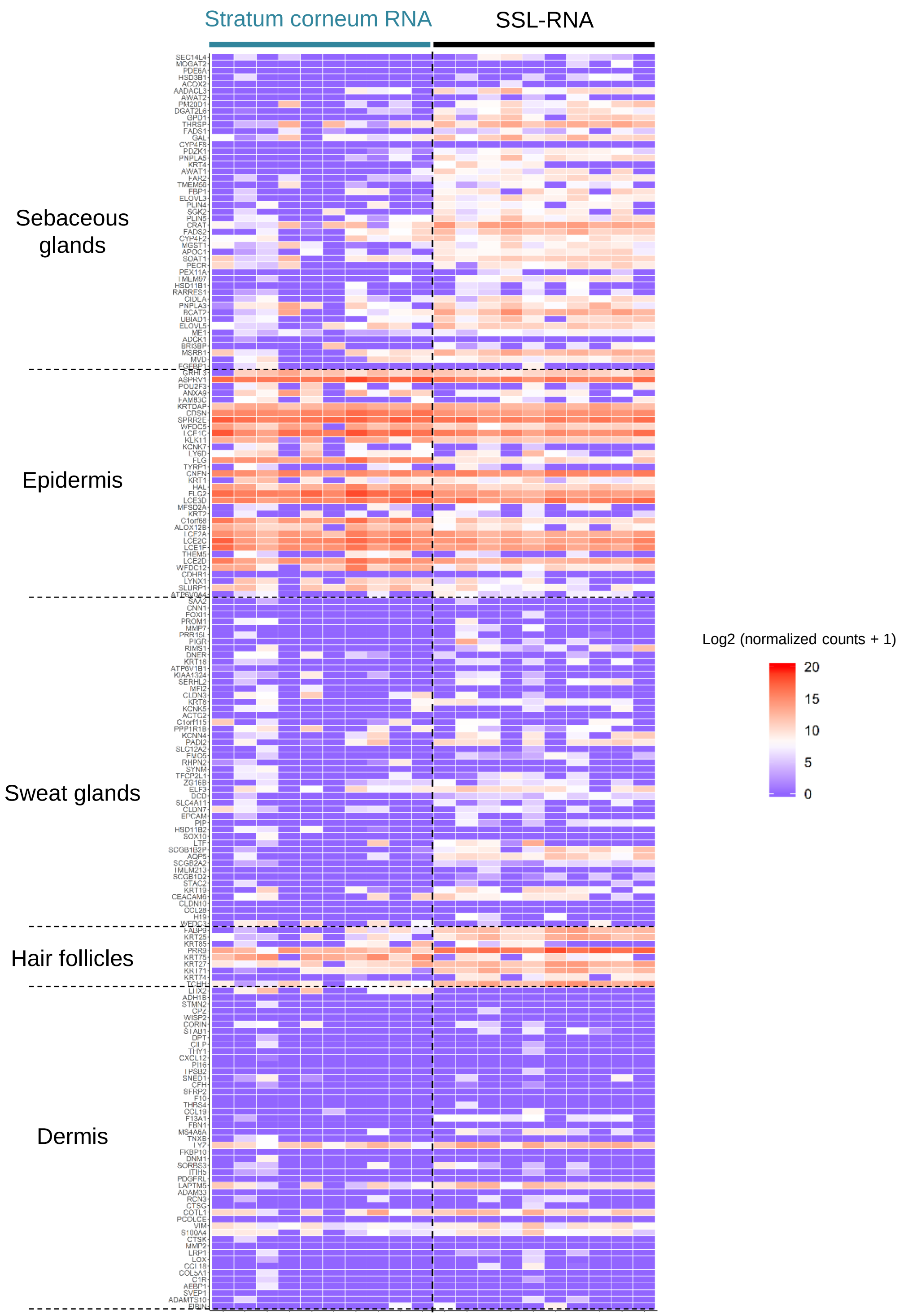

Supplementary Figure 2. mRNA expression in SSLs and stratum corneum.

SSL-RNAs and stratum corneum RNAs were collected from the same 10 healthy male subjects. The expression of genes characteristic of each skin region in SSL-RNAs and stratum corneum RNAs is shown in the heat map.

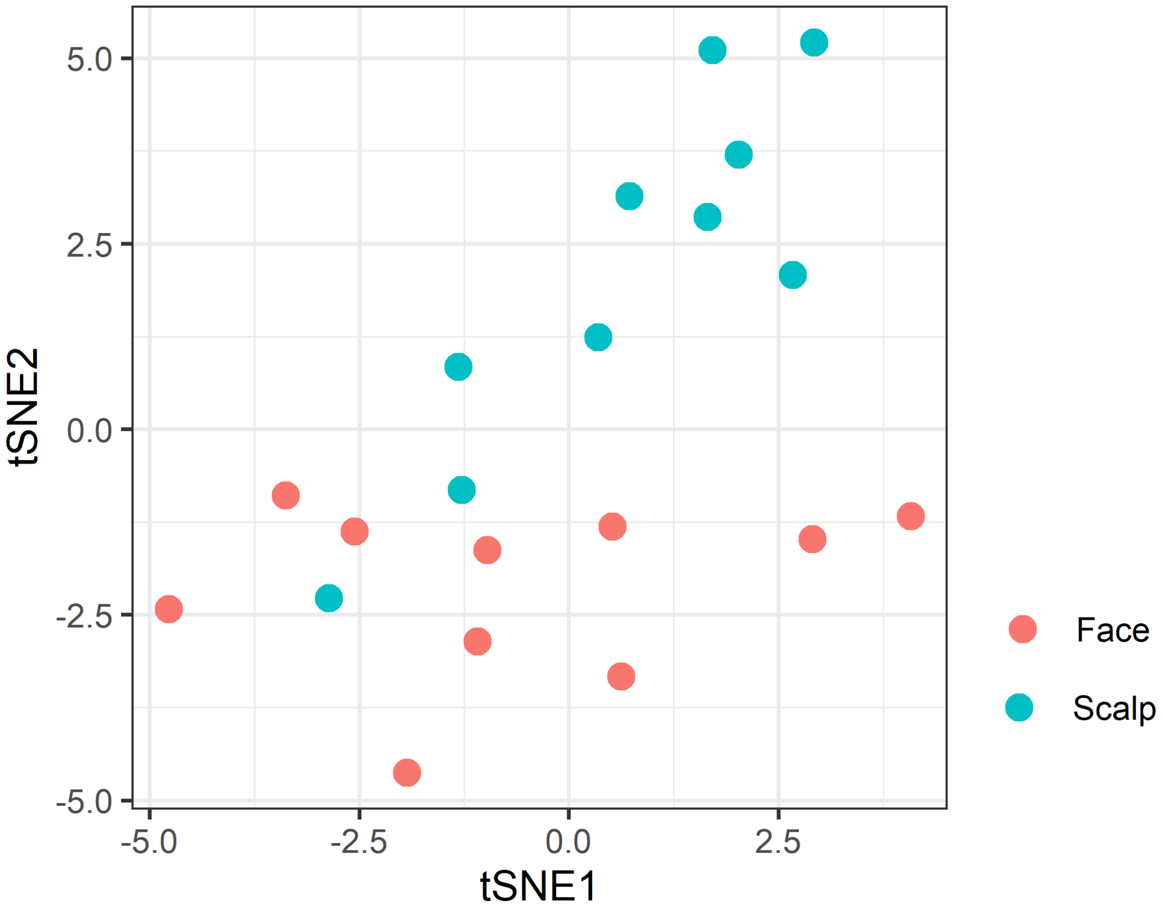

Supplementary Figure 3. Characterization of SSL-RNAs profiles in face and scalp. t-SNE analysis using variance stabilizing transformation (VST) values for all genes (red, face; green, scalp).

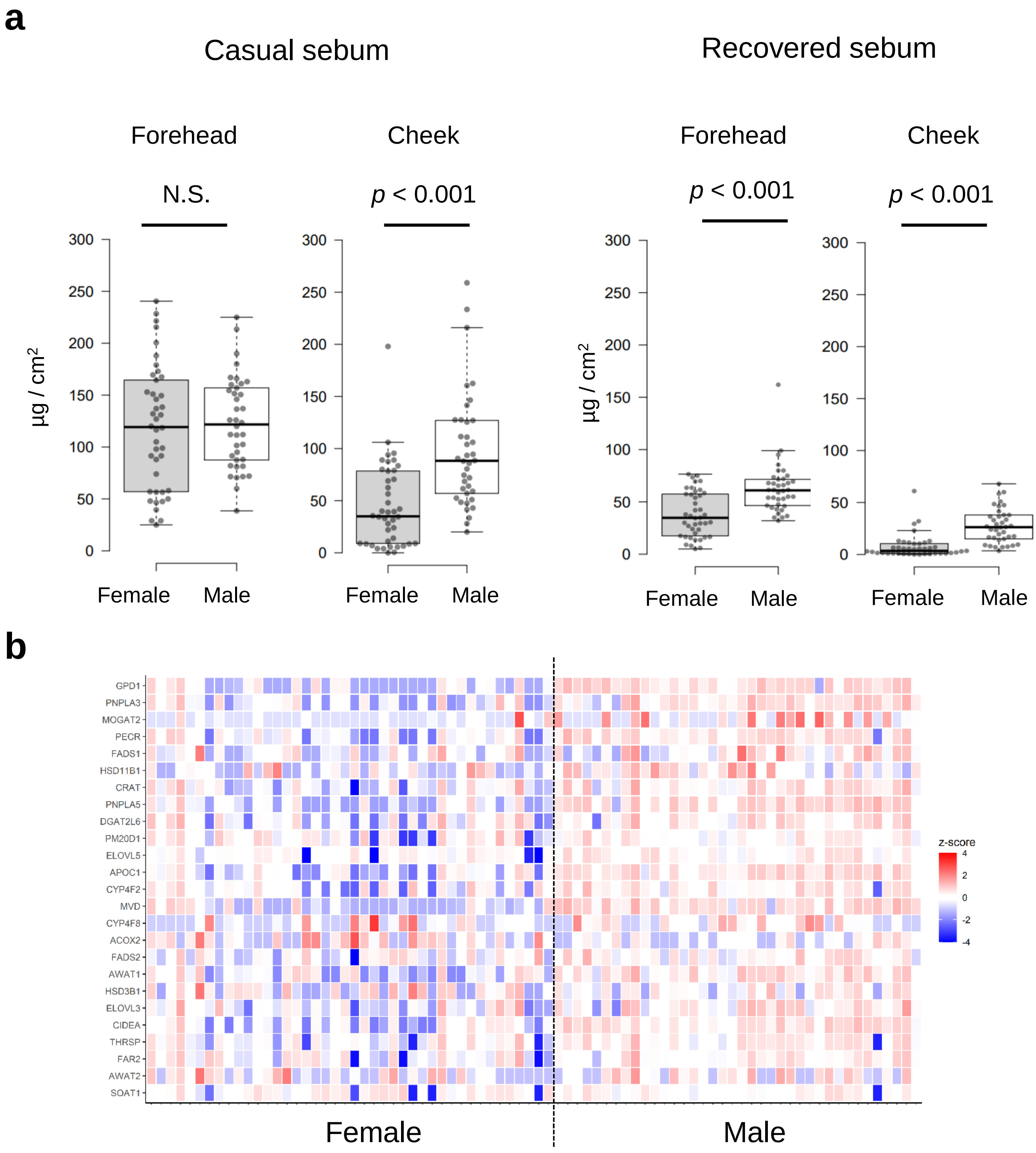

Supplementary Figure 4. Comparison the casual and the recovered sebum levels between males and females.

Facial sebum secretions (forehead and cheek) were measured using a Sebumeter. (a) The casual and recovered sebum levels in forehead and cheek. The level of significance of differences among the groups was analyzed using Student *t*-test. **p*<0.05, ***p*<0.01. (b) Heatmaps using z-transformed log2 (normalized counts + 1) on 25 lipid metabolism-related genes that were highly expressed in sebaceous glands (Fig. 7b). N.S.: not significant. Females (n = 42), males (n = 38).

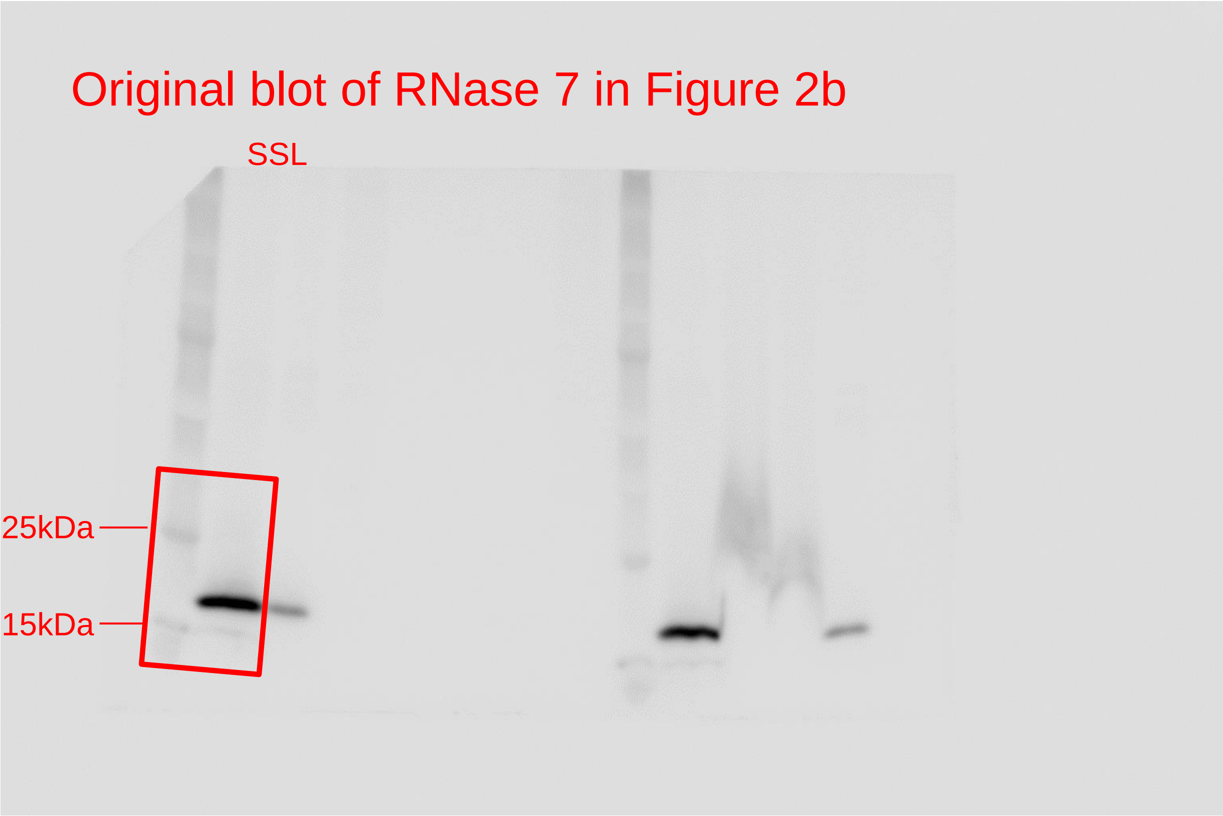

Supplementary Figure 5. Full image of blots

Supplementary Method 1: Detailed protocol for the SSL-RNA analysis

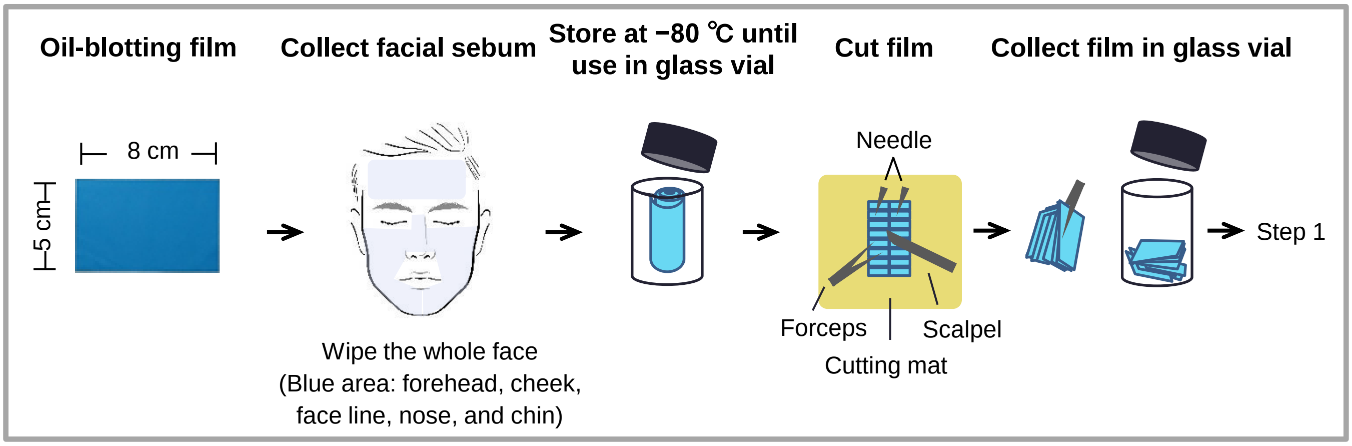

Extraction of RNA from facial skin surface lipids (SSLs)

1) Add 2.85 mL of QIAzol reagent to the glass vial, vortex, and transfer only the QIAzol solution to two fresh tubes.
2) Add 260 μL of chloroform to each tube, then vortex and centrifuge at 12,000 x *g* for 15 min at 4 ℃.

Purification and concentration of SSL-RNA

3) Transfer the upper layer (aqueous phase) to a fresh tube.
4) Purify total RNA using the RNeasy Mini kit (performing DNase treatment in the purification step) according to the manufacturer's instructions and elute from the resin twice with nuclease-free water (50 μL each time). Combine the eluates (100 μL each) into one tube (total 200 μL).
5) Concentrate total RNA by ethanol precipitation and finally dissolve in 10 μL of nuclease-free water.

Library preparation with AmpliSeq

6) Mix 1.75 μL of RNA solution with 0.5 μL of VILO reaction mix and 0.25 μL of SuperScript III Enzyme and perform reverse transcription under the following conditions: 25 ℃ for 10 min, 42 ℃ for 90 min, and then 85 ℃ for 5 min.
7) Mix 2.5 μL of cDNA solution, 1.5 μL of nuclease-free water, 2 μL of Ion AmpliSeq HiFi Mix, and 4.0 μL of Ion AmpliSeq Transcriptome Human Gene Expression Core Panel and amplify under the following conditions: 99 ℃ for 15 sec, 62 ℃ for 16 min, total 20 cycles (target amplification).
8) Purify the amplified DNA library with 10 μL of AMPure XP beads according to the manufacturer's instructions and then elute with 10 μL of nuclease-free water.
9) Check the quality of the DNA library with High Sensitivity D1000 ScreenTape and Agilent 4200 TapeStation. If the DNA library is amplified, a major band (approximately 170 bp in size) would be observed.
10) Mix 3.5 μL of purified DNA library solution, 2 μL of Ion AmpliSeq HiFi Mix, 4 μL of Ion AmpliSeq Transcriptome Human Gene Expression Core Panel, 0.5 μL of VILO reaction mix, and 1 μL of FuPa. Incubate the tube under the following conditions: 50 ℃ for 10 min, 55 ℃ for 10 min, and 60 ℃ for 20 min to partially digest the primer sequences.
11) Add 2 μL of Switch solution, 1 μL of Ion Xpress Barcode Adapters, and 1 μL of DNA Ligase to 11 μL of reaction solution, followed by incubation at 22 ℃ for 60 min and 72 ℃ for 5 min to ligate the adaptor sequences.
12) Purify the DNA library with 18 μL of AMPure XP beads and then elute and dissolve using 50 μL of Library Amp Mix.
13) After adding 2 μL of Library Amp Primers, perform library amplification under the following conditions: 98 ℃ for 15 s, 64 ℃ for 1 min, total 5 cycles.
14) Purify the amplified DNA library with 25 μL of AMPure XP beads and then transfer the supernatant to a fresh PCR tube. Further, purify the library again with 60 μL of AMPure XP beads and then elute with 10 μL of TE.
15) Check the quality of the DNA library with High Sensitivity D1000 ScreenTape and Agilent 4200 TapeStation. If the DNA library is amplified, a major band (approximately 230 bp in size) would be observed.

RNA-seq

16) Quantify the DNA library concentration using the Ion Library TaqMan Quantitation Kit.
17) After setting 50 pM DNA library in the Ion-chef system, perform emulsion PCR, template preparation, and chip loading.
18) Perform RNA-seq on the Ion-S5/X system.

Note: SSL-RNAs can be collected and analyzed from scalp, chest, back, and buttocks.

Supplementary Method 2. Library preparation for Ion AmpliSeq for comparing between SSL-RNA and stratum corneum RNA.

For extracting the RNA from SSL, a finely cut oil-blotting film containing sebum samples was treated with 1.45 mL of QIAzol reagent (Qiagen), after which the QIAzol solution was transferred to a new tube. Chloroform (260 μL) was added to the tube and the tube was vortexed and centrifuged at 12,000 × *g* for 15 min at 4 °C. The upper layer was transferred to a fresh tube and mixed with equal volumes of 85% ethanol. RNA was purified using the RNeasy mini kit (performing DNase treatment in the purification step) (Qiagen) and was eluted from the resin using a two-step process involving 50 μL of nuclease-free water. This was followed by ethanol precipitation, after which the RNA was dissolved in 10 μL of nuclease-free water.

For extracting RNA from the stratum corneum, RNeasy micro kit (Qiagen) was used; 600 μL of RLT buffer containing 40 mM DTT was added to extract RNA from the stratum corneum attached to the D-squames, and then the RLT solution was transferred to a tube and mixed with equal volumes of 85% ethanol. Subsequent purification steps were performed according to the manufacturer's protocol and RNA was eluted using 14 μL of nuclease-free water.

Then, 1.5 μL of RNA solution was mixed with 0.5 μL of VILO Reaction Mix, 0.25 μL

of SuperScript III Enzyme, and 0.25 μl of T4GP32 (concentration; 1 μg/μl) (NEB, Ipswich, MA, USA). Reverse transcription was performed using the following cycle: 25 °C for 10 min, 42 °C for 90 min, and finally 85 °C for 5 min. The PCR mix contained the following: 2.5 μL of cDNA, 1.4 μL of nuclease-free water, 2.0 μL of Ion AmpliSeq HiFi Mix, 4.0 μL of the Ion AmpliSeq Transcriptome Human Gene Expression Core Panel, and 0.1 μl of T4GP32 (concentration; 1 μg/μl) (NEB). PCR was performed using the following conditions: 99 °C for 15 s and 62 °C for 16 min for 20 cycles. The amplified DNA library was purified by mixing it with 10 μL of AMPure XP beads (Beckman Coulter, Miami, FL, USA) according to the manufacturer's protocol and then eluting it with 10 μL of nuclease-free water. The DNA library was quality checked using the High Sensitivity D1000 ScreenTape on the Agilent 4200 TapeStation. Upon amplification of the DNA library, a band of approximately 170 bp in size was observed. After checking the quality of the DNA library, the reaction solution was prepared by mixing 3.5 μL of purified library solution, 0.5 μL of the Ion AmpliSeq HiFi Mix and 5.5 μL of RNase free water, and 0.5 μL of the VILO Reaction Mix. After adding 1.0 μL of the FuPa reagent to 10 μL of the reconstituted reaction solution, the primer sequence was partially digested under the following conditions: 50 °C for 10 min, 55 °C for 10 min, and 60 °C for 20 min. To ligate the adaptor sequence, 2.0 μL of the Switch solution, 1.0

µL of the Ion Xpress Barcode adapters, and 1 µL of DNA ligase were added to 11 µL of reaction solution, followed by incubation at 22 °C for 60 min and 72 °C for 5 min. The library (15 µL) ligated with the adaptor sequence was purified by mixing with 18 µL of the AMPure XP beads according to the manufacturer's protocol. Libraries were eluted using 25 µL of the Library Amp Mix (Thermo Fisher Scientific) to which 1 µL of the Library Amp Primers were added. Library amplification was conducted using following cycle: 98 °C for 15 s and 64 °C for 1 min for five cycles. Next, 25 µL of the PCR product was mixed with 13 µL of the AMPure XP beads and the supernatant was transferred to fresh PCR tubes. The supernatants were mixed with 30 µL of the AMPure XP beads and purified; target fragments were eluted from the beads using 10 µL of the TE buffer. The library was quality checked using the High Sensitivity D1000 ScreenTape on the Agilent 4200 TapeStation.

Supplementary Table 1. The expressions of hair keratins and hair follicle-specific epithelial keratins in face and scalp.

Hair keratin (Type 2)

	Mean of $\log_2$ (normalized counts) + 1		FC (scalp / face)	FDR
	Face	Scalp		
KRT81	4.74	7.84	1.66	0.321
KRT82	1.06	6.40	6.02	3.54E-08
KRT83	2.97	6.29	2.12	0.429
KRT84	1.00	9.19	9.19	1.41E-16
KRT85	1.87	8.06	4.32	0.045
KRT86	5.91	5.18	0.88	0.880

Hair follicle-specific epithelial keratins (Type 1)

	Mean of $\log_2$ (normalized counts) + 1		FC (scalp / face)	FDR
	Face	Scalp		
KRT25	9.59	17.20	1.79	5.69E-16
KRT26	3.83	7.66	2.00	0.00777
KRT27	11.54	18.55	1.61	9.10E-39
KRT28	2.19	6.17	2.82	0.306

Hair follicle-specific epithelial keratins (Type 2)

	Mean of $\log_2$ (normalized counts) + 1		FC (scalp / face)	FDR
	Face	Scalp		
KRT71	11.50	17.75	1.54	2.63E-35
KRT72	9.50	16.55	1.74	1.94E-27
KRT73	1.00	5.92	5.92	3.50E-09
KRT74	4.67	13.34	2.85	4.78E-09
KRT75	4.00	9.59	2.40	0.353

FDR: Benjamini-Hochberg adjusted *p*-values are shown from the likelihood ratio test between face and scalp. Face (n = 10), scalp (n = 10). FC: fold change.
