# Supplementary material for: Non-invasive human skin transcriptome analysis using mRNA in skin surface lipids
Source: Commun Biol. 2022 Mar 9;5:215. doi: 10.1038/s42003-022-03154-w (PMC8907185; doi:10.1038/s42003-022-03154-w)
Supplement: Supplementary file 3 — Description of Additional Supplementary Files [file 42003_2022_3154_MOESM3_ESM.pdf]

Description of Additional Supplementary Files

File name: Supplementary Data 1
Description: The list of differentially expressed genes between the healthy subjects and the patients with AD.

File name: Supplementary Data 2
Description: Source data.
