# Supplementary material for: Non-invasive human skin transcriptome analysis using mRNA in skin surface lipids
Source: Commun Biol. 2022 Mar 9;5:215. doi: 10.1038/s42003-022-03154-w (PMC8907185; doi:10.1038/s42003-022-03154-w)
Supplement: Supplementary file 6 — Reporting Summary [file 42003_2022_3154_MOESM6_ESM.pdf]

nature research

Corresponding author(s): Takayoshi Inoue
Takatoshi Murase

Last updated by author(s): Dec 16, 2021

Reporting Summary

Nature Research wishes to improve the reproducibility of the work that we publish. This form provides structure for consistency and transparency in reporting. For further information on Nature Research policies, see our Editorial Policies and the Editorial Policy Checklist.

Statistics

For all statistical analyses, confirm that the following items are present in the figure legend, table legend, main text, or Methods section.

n/a	Confirmed	
☐	☒	The exact sample size (*n*) for each experimental group/condition, given as a discrete number and unit of measurement
☐	☒	A statement on whether measurements were taken from distinct samples or whether the same sample was measured repeatedly
☐	☒	The statistical test(s) used AND whether they are one- or two-sided<br>*Only common tests should be described solely by name; describe more complex techniques in the Methods section.*
☒	☐	A description of all covariates tested
☐	☒	A description of any assumptions or corrections, such as tests of normality and adjustment for multiple comparisons
☐	☒	A full description of the statistical parameters including central tendency (e.g. means) or other basic estimates (e.g. regression coefficient) AND variation (e.g. standard deviation) or associated estimates of uncertainty (e.g. confidence intervals)
☐	☒	For null hypothesis testing, the test statistic (e.g. F, t, r) with confidence intervals, effect sizes, degrees of freedom and P value noted<br>*Give P values as exact values whenever suitable.*
☒	☐	For Bayesian analysis, information on the choice of priors and Markov chain Monte Carlo settings
☒	☐	For hierarchical and complex designs, identification of the appropriate level for tests and full reporting of outcomes
☒	☐	Estimates of effect sizes (e.g. Cohen's *d*, Pearson's *r*), indicating how they were calculated

Our web collection on statistics for biologists contains articles on many of the points above.

Software and code

Policy information about availability of computer code

Data collection	No software was used to collect the data in this study.
Data analysis	We used publicly available softwares for the analysis. Software: R (v3.6.1), DESeq2 (v1.24.0), Rtsne (v0.15), Torrent Suite Software (v5.2.1)

For manuscripts utilizing custom algorithms or software that are central to the research but not yet described in published literature, software must be made available to editors and reviewers. We strongly encourage code deposition in a community repository (e.g. GitHub). See the Nature Research guidelines for submitting code & software for further information.

Data

Policy information about availability of data

All manuscripts must include a data availability statement. This statement should provide the following information, where applicable:

- Accession codes, unique identifiers, or web links for publicly available datasets
- A list of figures that have associated raw data
- A description of any restrictions on data availability

The datasets generated and analyzed in the current study are available from the corresponding author on reasonable request.
Accession codes will be available before publication.

Field-specific reporting

Please select the one below that is the best fit for your research. If you are not sure, read the appropriate sections before making your selection.

☒ Life sciences ☐ Behavioural & social sciences ☐ Ecological, evolutionary & environmental sciences

For a reference copy of the document with all sections, see nature.com/documents/nr-reporting-summary-flat.pdf

Life sciences study design

All studies must disclose on these points even when the disclosure is negative.

Sample size	No sample-size calculation was performed. Sample sizes were decided based on previous publications (PMID: 21388663, PMID: 22951056), experience and common standards in similar field for calculating statistical significance and also dependent on sample availability.
Data exclusions	We collected sebum from 133 subjects in the study. However, SSL-RNA samples collected from 3 subjects were excluded because those samples did not meet the manufacturer's criteria of AmpliSeq library preparation.
Replication	The reproducibility of our established transcriptome method for SSL-RNA was described in the text.<br>Data were not replicated to collect SSL from whole face or scalp at one time.<br>The SSL-RNA comparison study (AD vs healthy, face vs scalp and high levels of sebum vs low levels of sebum) was performed only once.
Randomization	Random allocation was not relevant to our study because we assigned samples as groups based on skin condition (AD vs healthy) diagnosed by the dermatologist.
Blinding	There was no blinding in this study because subjects were allocated based on the dermatologist's examination, there were no treated or untreated groups, and intervention bias was not an issue given the nature of the study. Hierarchical clustering and statistical analyses performed following transcriptome analysis are inherently unbiased approaches.

Reporting for specific materials, systems and methods

We require information from authors about some types of materials, experimental systems and methods used in many studies. Here, indicate whether each material, system or method listed is relevant to your study. If you are not sure if a list item applies to your research, read the appropriate section before selecting a response.

Materials & experimental systems

n/a	Involved in the study	
☐	☒	Antibodies
☒	☐	Eukaryotic cell lines
☒	☐	Palaeontology and archaeology
☒	☐	Animals and other organisms
☐	☒	Human research participants
☒	☐	Clinical data
☒	☐	Dual use research of concern

Methods

n/a	Involved in the study	
☒	☐	ChIP-seq
☒	☐	Flow cytometry
☒	☐	MRI-based neuroimaging

Antibodies

Antibodies used	RNase 7, poly-clonal; raised in rabbit; Cloud-Clone Corp.(Cat#: PAD193Hu01)<br>keratin/cytokeratin, mono-clonal; raised in mouse; Nichirei (Cat#: 412811, AE-1/AE-3)<br>rabbit IgG, poly-clonal (Alexa Fluor 555); raised in donkey; Thermo Fisher Scientific (Cat#: A31572)<br>mouse IgG, poly-clonal (Alexa Fluor 647); raised in goat; Thermo Fisher Scientific (Cat#: A21235)<br>rabbit IgG, monoclonal (horseradish peroxidase-linked secondary antibody; raised in donkey; GE Healthcare (Cat#: NA9340V)
Validation	Cat#: PAD193Hu01: Selected validation data available on supplier website.<br>Cat#: 412811, AE-1/AE-3: Selected validation data available on supplier website.<br>Cat#: A31572: Selected validation data available on supplier website.<br>Cat#: A21235: Selected validation data available on supplier website.<br>Cat#: NA9340V: Selected validation data available on supplier website.

Human research participants

Policy information about studies involving human research participants

Population characteristics	Skin condition of healthy individuals or patients with AD was diagnosed by the dermatologist.<br>1) Healthy subjects: 32 healthy male individuals (mean age: 34.6 years, range: 20–49 years)

	2) Patients with AD: 30 male patients (mean age: 31.0 years, range: 20–48 years)<br>Sebum level in males and females was measured by sebumeter ((SM815, C-K Electronics, Cologne, Germany).<br>3) Male subjects: 41 healthy male individuals (mean age: 39.1 years, range: 20–58 years) involving 32 subjects of 1)<br>4) Female subjects: 42 healthy female individual (mean age: 38.5 years, range: 20–56 years)<br>Comparing SSL-RNAs and stratum corneum RNAs<br>5) 10 healthy male individuals (mean age: 40.6 years, range: 28–57 years)<br>Comparing SSL-RNA profile between face and scalp<br>6) 10 healthy female individuals (mean age: 26.3 years, range: 24–32 years)
Recruitment	Subjects with self-reported healthy skin or AD were recruited, and include those subjects after diagnosis of skin condition by dermatologist.
Ethics oversight	The study was approved by the Human Research Ethics Committee, Kao Corporation (approval numbers: approval numbers: 792-2016082, T003-170413, T173-180920 and T246-190815), Japan Aesthetic Dermatology Symposium (approval number: KU-2017-05-003), the Shinjukuminamiguchi Dermatologic Clinic (approval number: KU-2016-10-005), and the Institutional Review Board of IntegReview Ltd. (Austin, TX, USA; approval number: T046a-170829). All participants proved written informed consent.

Note that full information on the approval of the study protocol must also be provided in the manuscript.
